# Healthcare professionals’ ethical competence: A scoping review

**DOI:** 10.1002/nop2.173

**Published:** 2018-07-16

**Authors:** Janika Koskenvuori, Minna Stolt, Riitta Suhonen, Helena Leino‐Kilpi

**Affiliations:** ^1^ Department of Nursing Science University of Turku Finland; ^2^ Turku University Hospital Finland; ^3^ City of Turku, Welfare Division Finland

**Keywords:** competence, ethical competence, ethics, healthcare professionals, moral, scoping review

## Abstract

**Aim:**

The aim of this study was to examine the extent and nature of the available research literature on healthcare professionals’ ethical competence and to summarize the research findings in this field.

**Design:**

A scoping review guided by Arksey and O'Malleys methodological framework was conducted.

**Methods:**

Six databases including Pubmed/Medline, CINAHL, Web of Science Core Collection, PsycInfo, Philosophers’ Index, and Scopus were searched systematically. Of 1,476 nonduplicate citations, 17 matched the inclusion criteria.

**Results:**

Findings revealed that healthcare professionals’ ethical competence is a limited but topical research area. The focus areas of the studies were conceptualization, measuring, and realization of the ethical competence. The studies provided varying definitions and constructions for ethical competence and a few instruments to measure ethical competence were identified. Research in this area seems to be in a transition phase from theorization to empirical measurement. Methodologically, the research was rather heterogeneous and mainly focused on nurses.

## 1 INTRODUCTION

In recent decades, ethical demands on healthcare professionals have increased due to factors such as scarce resources (Kälvemark, Höglund, Hansson, Westerholm, & Arnetz, 2004), need for prioritization (de Groot et al., 2017) and improved medical and technological advances which expand treatment and care options (Fleck, 2013). To meet this development, different ethical codes and guidelines have been developed to guide healthcare professionals’ behaviours and actions (Dahnke, 2014; Numminen, Arend, & Leino‐Kilpi, 2009). Furthermore, ethics courses have been included in curricula for healthcare professionals, and ethical committees, ethical rounds, and educations have been implemented into healthcare organizations to support healthcare professionals in handling ethically demanding situations (Chao, Chang, Yang, & Clark, 2017; Molewijk, Zadelhoff, Lendemeijer, & Widdershoven, 2008). Such guidance, education, and support are reasonable as healthcare professionals have an important role and considerable responsibility in analysing and resolving ethical issues encountered in their daily practice (Rees, Lindy, & Schmitz, 2009). To achieve this, being ethically competent is a necessity.

Theoretical understanding of the concept of ethical competence seems to vary. Ethical competence, also referred to as moral competence, is considered as one component of professional competence (Jormsri, Kunaviktikul, Ketefian, & Chaowalit, 2005; Paganini & Egry, 2011) consisting of the knowledge, skills, and attitudes required to address ethical issues (Robichaux, 2016). According to another consideration, ethical competence consists of a moral agent's ability to identify value conflicts and ethical dimensions, ability to choose one value over another based on logical reasoning, and ability to act based on the judgement that has been performed. (Jormsri et al., 2005). Furthermore, ethical competence has been seen as a matter of being (personal characteristics), doing (acting according to the judgements made based on the principles and rules), and knowing (being familiar with the ethical laws and guidelines) (Eriksson, Helgesson, & Höglund, 2007). Despite the somewhat differing terminology used in the definitions, there seems to be an understanding that ethical competence is a crucial factor enabling healthcare professionals to make complex, value‐based decisions and to implement ethically sustainable care (Clark & Taxis, 2003) and hence, to restrain mistreatment and ethically obscure actions in healthcare. (Bolmsjö, Sandman, & Andersson, 2006; Nordström & Wangmo, 2017).

Given the importance of healthcare professionals’ ethical competence in providing quality and good patient care with respect to patients’ rights, a scoping review was conducted. The objective of this scoping review was to examine the extent and nature of the available research literature on healthcare professionals’ ethical competence and to summarize the research findings in this field. We identified gaps in the evidence base where no or limited research has been conducted and point out the needs for further research. This knowledge is valuable, especially for researchers. A variety of definitions for ethical competence have been given in the literature, and we did not refer to any sole definition. Instead, we wanted to include a variety of scope in the relevant literature focusing on ethical competence of healthcare professionals.

## 2 METHODS

This scoping review was guided by Arksey and O'Malley's (2005) methodological framework including five different stages: (a) identifying the research questions, (b) identifying relevant studies, (c) study selection, (d) charting the data, and (e) collating, summarizing, and reporting the results. Scoping review is an increasingly popular literature review method, especially in healthcare research, allowing researchers to map a specific research field for relevant research literature with broad research questions to summarize research findings and find gaps in the research field in question (Arksey & O'Malley, 2005).

To answer the research objective, the authors *identified the research questions* as follows:

1. What are the focus areas of the studies?
2. What are the research findings?
3. What are the methodological orientations of the studies?
4. How are the reliability and validity assessments of the studies addressed?

For *identifying relevant studies*, the following databases were searched from their earliest: Pubmed/Medline (1966)*, *CINAHL (1988)*, *Web of Science Core Collection (1900), PsycInfo (1880), Philosophers’ Index (1967), and Scopus (1970). Unlabelled search query “(ethical competenc*) OR (ethical skill*) OR (moral competenc*) OR (moral skill*)” with the English language limitation was used. A database search without any time limitations was run in March 2017 yielding 1,476 citations after duplicate removal.

*Study selection* was carried out against the predefined inclusion criteria, which were: (a) empirical study including concept analyses and literature reviews; (b) published in scientific referee‐based journal and (c) on healthcare professionals’ ethical competence, (d) in healthcare context. Studies were excluded when (a) the informants were solely students or (b) the paper was unempirical or theoretical.

**Figure 1:**
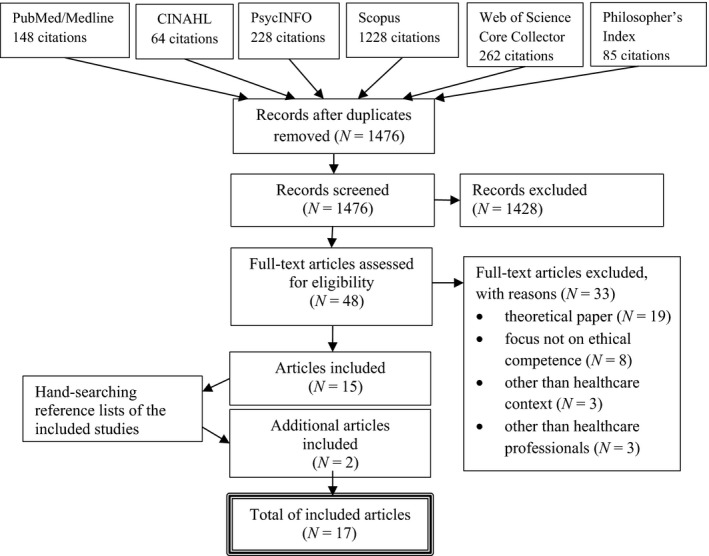
Scoping review flowchart

The study retrieval process was conducted independently by two reviewers (JK & MS) by applying inclusion and exclusion criteria to all citations by title and abstract to identify studies meeting the research objective. If the pertinence of the citation was obscure, the full text of the study was obtained and reviewed. After discussion and consensus decision‐making, 48 articles were selected for full‐text analysis. All full texts were obtained, reviewed, and selected independently by JK and MS. Any disagreements on selecting the studies were resolved by discussion and confirmed within the research team resulting in 15 studies to be included in this review. In addition, reference lists of included studies were handsearched for other potential articles, yielding two more studies and thus, altogether 17 studies to be included (Figure 1). Critical appraisal was conducted for all included studies by two members of the research team (JK, MS or HL‐K) for each of the studies using the Joanna Briggs Institute appraisal tools (Joanna Briggs Institute, 2017) to assess the methodological quality of the studies as well as to determine the extent to which the studies had addressed the possibility of biases. However, the function of the critical appraisal was not selective but rather descriptive.

For *charting the data*, a predeveloped data charting form consisting of descriptive study characters (authors, year, journal, location, study aim, setting, study design, informants, sampling method, sample size, data collection method, data analysis, validity and reliability discussion, limitations, and results) was used. Data were charted by JK, MS, and HL‐K.

*Collating, summarizing, and reporting the results* were carried out in accordance with the research questions using narrative approach and self‐tailored thematic constructions. Furthermore, some quantification was made. Expressions used in the original studies were used and no interpretations were made.

## 3 ETHICS

As this was a scoping review, ethical approval was not required. The review was conducted according to good scientific integrity.

## 4 RESULTS

### 4.1 General description of the studies

The studies were conducted within a 12‐year range as the first one was published in 2004. The number of publications remained quite low until the year 2010 as only two earlier publications emerged. During the time period 2010–2014, there were one or two publications each year, after which the interest in ethical competence started to increase. Half of the studies were published in 2015 (*N* = 3) and 2016 (*N* = 6).

Most of the studies were conducted in Western Europe, including Finland (*N* = 4), the Netherlands (*N* = 3), Sweden (*N* = 2), and Portugal (*N* = 1). The North American studies were conducted in Canada (*N* = 2) and the USA (*N* = 1) and the Far Eastern studies in Japan (*N* = 2) and Thailand (*N* = 1). One study was conducted in the Middle Eastern country of Iran (*N* = 1).

Majority of the studies used a multisite setting containing different clinical areas or healthcare contexts (*N* = 8) (Cusveller, 2012; Cusveller & Schep‐Akkerman, 2016; Falkenström, Ohlsson, & Höglund, 2016; Höglund, Eriksson, & Helgesson, 2010; Jormsri, Kunaviktikul, Chaowalit, & Ketefian, 2004; Poikkeus, Numminen, Suhonen, & Leino‐Kilpi, 2014; Poikkeus, Suhonen, Katajisto, & Leino‐Kilpi, 2016; Schaefer & Vieira, 2015). One‐site settings included public health (*N* = 1) (Asahara, Kobayashi, & Ono, 2015), home care (*N* = 1) (Asahara et al., 2015), psychiatric hospital (*N* = 1) (Molewijk, Verkerk, Milius, & Widdershoven, 2008), aggressive care (*N* = 1) (Peter, Mohammed, & Simmonds, 2015), and academic (*N* = 1) (Chambers, 2011) settings. One study used a combination of clinical and educational settings (Barkhordari‐Sharifabad, Ashktorab, & Atashzadeh‐Shoorideh, 2016).

### 4.2 Focus areas

Three main focus areas were identified as follows: (a) conceptualization, (b) measuring, and (c) realization of the ethical competence. The first focus area of the studies had a conceptual approach as they were defining (Jormsri et al., 2004; Kulju, Stolt, Suhonen, & Leino‐Kilpi, 2016), analyzing (Kulju et al., 2016), and exploring the construct of the concept of ethical competence (Lechasseur, Caux, Dollé, & Legault, 2016). The studies also focused on describing healthcare managers’ (Barkhordari‐Sharifabad et al., 2016) and research nurses’ ethical competencies (Höglund et al., 2010) as well as on the competences needed in participation in ethics committees (Cusveller, 2012) and meetings (Cusveller & Schep‐Akkerman, 2016).

The second focus area of the studies was measuring ethical competence. The studies developed and evaluated new instruments to measure healthcare professionals’ ethical competence in terms of moral competence (Asahara et al., 2015; Asahara, Ono, Kobayashi, Omori, & Todome, 2013; Jormsri et al., 2004) and moral skills (Chambers, 2011). Measuring the level of ethical competence and perceptions of support for it have also been focal points of studies (Poikkeus et al., 2016; Poikkeus, Numminen, et al., 2014).

The third focus area of the studies was the realization of ethical competence as they explored the role of ethical competence in coping with moral distress (Schaefer & Vieira, 2015), fostering hope (Peter et al., 2015) and handling conflicts of interest (Falkenström et al., 2016). Furthermore, the studies have focused on moral case deliberation in terms of improving ethical competence (Molewijk, Verkerk, et al., 2008) and on supporting nurses’ ethical competence (Poikkeus, Leino‐Kilpi, & Katajisto, 2014).

### 4.3 Research findings

The research findings are summarized and presented in accordance with the previously identified main focus areas: (a) conceptualization, (b) measuring, and (c) realization of the ethical competence. The conceptualization of ethical competence varied among the studies as they provided differing definitions and constructions for the phenomenon. Ethical competence was defined in terms of moral competence as “the ability or capacity of persons to recognize their feelings when they encounter what is morally right or wrong in particular situations and then to reflect on those feelings to direct their decisions and act in ways which bring about the highest level of benefaction for patient's best interest.” (Jomsri et al., 2004). Another definition was provided through the concept analysis process where the ethical competence was defined in terms of character strength, ethical awareness, moral judgement skills and willingness to do good. According to the same analysis, the prerequisites for ethical competence were “virtuous professional, experience of a professional, human communication, ethical knowledge and supporting surroundings in the organization.” The consequences of ethical competence were “the best possible solutions for the patient, reduced moral distress at work and development and democratization of society” (Kulju et al., 2016). The construct of ethical competence was composed of ethical sensitivity, ethical knowledge, ethical reflection, ethical decision‐making, ethical action, and ethical behaviour (Lechasseur et al., 2016).

The conceptualization of ethical competence also varied in different proficiency contexts. The main categories of nurse leaders’ ethical competency were labelled as empathetic interactions, ethical behaviour, and exalted manners (Barkhordari‐Sharifabad et al., 2016). Among research nurses, ethical competence was seen as character building, related to virtues such as being honest, empathic, and loyal to patients. Furthermore, ethical competence was assumed to be learnt through role models, good examples, and practical experience, while ethical guidelines were not perceived as valuable in this process (Höglund et al., 2010). The competencies that nurses needed in participating in ethics committees were reported through knowledge, skills, and attitudes. Knowledge was regarded through health law, ethics, and professional knowledge. Regarding skills, communication was mentioned, as were the professional skills and skills for “doing ethics.” Regarding attitude, an open and respectful attitude toward patients and fellow committee members and commitment to patient care, work, and professional ethics were required. (Cusveller, 2012.) This competency profile regarding competencies needed in participating in ethics committees was further established in a latter study (Cusveller & Schep‐Akkerman, 2016).

For measuring ethical competence, four different instruments were identified. The instruments measuring nurses’ ethical competence were Moral Competence Scale for Home Care Nurses (MCSHCN) (Asahara et al., 2013), Moral Competence Questionnaire for Public Health Nurses (MCQ‐PHN) (Asahara et al., 2015), and Moral Competence Scale (MCS) (Jormsri et al., 2004). Item and explorative factor analysis (EF) for MCSHCN revealed 45 items loading on five factors. Confirmatory factor analysis (CF) was used to indicate that this five‐factor model had a reasonable fit to data and thus, corresponded closely to the theoretical components of moral competence. Cronbach's alphas for MCSHCN ranged from 0.85‐0.91 (Asahara et al., 2013.). EF for MCQ‐PHN revealed 15 items loading on three factors. This three‐factor model also showed reasonable fit to the data by CF. Cronbach's alphas for MCQ‐PHN ranged from 0.78 to 0.93 (Asahara et al., 2015.). MCS was derived from nursing values based on Thai culture and it included 108 items presented in five scenarios. MCS was preliminarily tested twice using small samples of nurses. The results showed that Cronbach's alpha for MCS was 0.77. With its content validity and reliability, the MCS was considered acceptable for further testing to obtain other psychometric properties (Jormsri et al., 2004). Nurses were not the only profession group having an instrument to measure ethical competence as Chambers (2011) developed and validated the Moral Skills Inventory for use in dentistry to measure the elements of Rest's four‐component model of moral behaviour. The long version of the instrument included 40 items and the short version 16 items. The results supported the use of the short version of the Moral Skills Inventory. However, further development work was needed before the use of this instrument could be recommended (Chambers, 2011).

As a part of measuring, estimation of the level of ethical competence was self‐assessed by nurses and nurse leaders. Nurses estimated their own ethical competence to be at an average level, whereas nurse leaders estimated their own competence to be at a high level. Nurses’ and nurse leaders’ perceptions of support provided for nurses’ ethical competence was not at a high level (Poikkeus et al., 2016). However, nurse leaders supported nurses’ ethical competence more often during performance reviews than during recruitment (Poikkeus, Numminen, et al., 2014).

The realization of ethical competence appeared through other substances. More specifically, it was studied how ethical competence posed itself as a coping resource, hope sustainment and when handling conflicts of interests. Ethical competencies as resources used by professionals to cope with moral distress were divided into positive and negative resources. Positive resources included discussion with work colleagues, holding meetings, reflection, learning from earlier experiences, changes in protocols, creativity and collective actions. Negative ones included avoiding ethically difficult situations, nondiscussion or reflection of the case and the option to change jobs (Schaefer & Vieira, 2015). Nurses’ moral competence related to fostering hope in patients and their families was identified as “Reimagining hopeful possibilities,” “Exercising caution within the social–moral space of nursing” and “Maintaining nurses’ own hope” (Peter et al., 2015). Among healthcare managers, ethical competence was of great importance to identify and handle conflicts of interest, consisting of contextual understanding, rational emotions, some theoretical knowledge and suitable language. They also found that top management needs to express the importance of ethical competence and allocate resources to allow adequate learning processes (Falkenström et al., 2016).

Enhancement and improvement of ethical competence were also addressed. Colleagues and nurse leaders have a key role in providing opportunities for nurses to enhance their ethical competence (Poikkeus, Leino‐Kilpi, et al., 2014). Healthcare professionals’ moral competencies (i.e., knowledge, attitude, and skills) could be improved through moral case deliberations (Molewijk, Verkerk, et al., 2008).

### 4.4 Methodological orientations

#### 4.4.1 Research designs

The studies were conducted using varying research designs, none of them being clearly predominant. Seven (41%) studies were quantitative, using a descriptive design. Five (29%) studies were qualitative, and they used descriptive (*N* = 3), explorative (*N* = 2), and critical qualitative approach (*N* = 1) designs. One (6%) study was a mixed‐methods study, using an interactive responsive evaluation design. Of all studies, three (18%) were literature reviews, four (24%) were instrument development and validation studies and one (6%) was concept analysis (Table 1).

**Table 1:** Methodological orientations of the studies

	Study aim	Study design	Sampling (RR%)	Data collection	Data analysis
Quantitative studies					
Jormsri et al. (2004); Thailand	To describe the development of the moral competence concept in nursing practice that is relevant to Thai nursing values and to present the construction of the Moral Competence Scale (MCS) for measuring moral competence in nursing practice.	Instrument development and validation Descriptive	1. Convenience Nurses *N* = 13 2. Purposive Nurses *N* = 33	Moral Competence Scale (MCS)/Jormsri et al. (2004)	Descriptive, Cronbach's alpha
Chambers (2011); USA	To describe the development and validation of a short paper‐and‐pencil instrument that can be self‐scored for use in dentistry to measure the elements of Rest's four‐component model of moral behaviour.	Instrument development and validation Descriptive	Dental students *N* = 196 clinical faculty members *N* = 41 regents and officers *N* = 19	Moral Skills Inventory (MSI)/Chambers (2011)	Descriptive, Pearson's correlation, Cronbach's alpha
Asahara et al. (2013); Japan	To describe the development of the Moral Competence Scale for Home Care Nurses and to evaluate its validity and reliability in Japan.	Instrument development and validation Descriptive	Stratified home care nurses *N* = 1989 (23.1%)	Moral Competence Scale for Home Care Nurses (MCSHCN)/Asahara et al. (2013)	Descriptive, CFA, EFA with maximum likelihood factor analysis and Promax rotation, Cronbach's alpha
Poikkeus, Numminen, et al., 2014; Finland	To analyse how nurse leaders support the ethical competence of nurses during recruitment and performance reviews	Descriptive	Purposive nurse leaders *N* = 198 (37%)	electronic online survey, structured questionnaire (new): 3 parts (background, support for ethical competence during recruitment, support for ethical competence of nurses during performance reviews)	Descriptive, Wilcoxon two‐sample test, Pearson's correlation, Pearson's chi‐square test, Cronbach's alpha, one‐way analysis of variance, multiple comparisons using either Tukey's or Tamhane's test.
Asahara et al. (2015); Japan	To develop a valid and reliable moral competence self‐assessment questionnaire for PHN that is easy to use in practice.	Instrument development and validation Descriptive	Public health nurses *N* = 3,409 (31.9%)	Moral Competence Questionnaire for Public Health Nurses (MCQ‐PHN)/Asahara et al. (2015)	Descriptive, Pearson's correlation, CFA, EFA, Cronbach's alpha
Cusveller and Schep‐Akkerman (2016); the Netherlands	To corroborate an existing profile of the requisite knowledge, skills, and attitudes in the form of a questionnaire contributes to the development of a tool to determine the competence nurses need for ethics meetings	Descriptive	Subscribers of the digital newsletter of three widely read nursing journals in the Netherlands *N* = 49	Questionnaire (developed for this study)	Descriptive
Poikkeus et al. (2016); Finland	To analyse the level of nurses’ and nurse leaders’ ethical competence, perceptions of support for nurses’ ethical competence at the organizational and individual levels and background factors associated with this support.	Descriptive	Systematic Nurses *N* = 298 (26%) Nurse leaders *N* = 193 (16%)	Ethical Competence questionnaire/Poikkeus et al. (2016) Ethical Competence Support questionnaire (EthiCS)	Descriptive, multifactor analysis of variance, *t* test
Qualitative studies					
Höglund et al. (2010); Sweden	To describe and explore the perception of ethical guidelines and their role in ethical competence building among Swedish physicians and research nurses.	Descriptive Explorative	Purposive research nurses *N* = 6	in‐depth interviews	Stepwise categorization method as by Malterud
Cusveller (2012); the Netherlands	The inquiry aimed at a preliminary competency description for participation in ethics committees. The article reports the aggregate of 52 interviews in five different studies.	Descriptive	Nurses *N* = 52	semistructured interview	Five original studies used inductive data analysis proposed by Baarda et al. Not mentioned how the results of these five studies were aggregated for this study.
Peter et al. (2015); Canada	To explore nurses’ moral competence related to fostering hope in patients and families within the context of aggressive technological care and to understand how competence is shaped in this environment.	Critical qualitative approach	Purposive graduate nursing students *N* = 15	semistructured interviews	Critical approach in all steps Content analysis, categorization Retroductive process
Barkhordari‐Sharifabad et al. (2016); Iran	To determine the ethical competency of nurse leaders in the Iranian cultural context and working conditions of the Iranian healthcare system.	Descriptive	Purposive nurse leaders *N* = 14	semistructured interview	Content analysis with deductive approach
Falkenström et al. (2016); Sweden	To explore what kind of ethical competence healthcare managers need in handling conflicts of interest (COI). The aim is also to highlight essential learning processes to develop healthcare managers’ ethical competence.	Explorative	Strategic healthcare managers *N* = 10	Semistructured interviews, each participant was interviewed twice except one.	A stepwise method
Mixed‐methods studies					
Molewijk, Verkerk, et al., (2008); the Netherlands	To (a) describe the practice and the theoretical background of moral deliberation, (b) describe the moral deliberation project, (c) present the outcomes of the evaluation of the moral case deliberation sessions, and (d) present the implementation process.	Interactive responsive evaluation design	healthcare professionals *N* = 69 (73%) healthcare professionals *N* = 49 (40%) healthcare professionals *N* **= ? healthcare professionals *N *= ?	Quantitative section: Maastricht evaluation questionnaires evaluation survey Qualitative section: in‐depth interviews, ethnographic participant observation	Quantitative data: Descriptive Qualitative data: Qualitative explorative analysis
Literature reviews and Concept analysis					
Poikkeus, Leino‐Kilpi, et al., 2014; Finland	To appraise and synthesize evidence of empirical studies of how nurses’ ethical competence can be supported.	Mixed‐method systematic review according to the University of York's Centre for Reviews and Dissemination guidelines	Empirical studies *N* = 34		Content analysis
Schaefer and Vieira (2015); Portugal	To seek evidence on ethical situations experienced by nurses; to identify the coping resources which they use; and to ascertain the role of ethical competence in coping with moral distress.	Integrative literature review	Empirical studies *N* = 23		n/a
Kulju et al. (2016); Finland	To report an analysis of the concept of ethical competence.	Concept analysis	Theoretical articles *N* = 12 Empirical articles *N* = 6		An entity theoretic strategy based on Wilson's method and modified by Walker and Avant was employed.
Lechasseur et al. (2016); Canada	To clarify this point in addition to better defining ethical competence in the context of nursing practice.	Integrative literature review	Scientific studies *N* = 35 Literature reviews *N* = 10 Theoretical articles *N* = 44		n/a

#### 4.4.2 Participants

The study participants were mainly nurses. In quantitative studies, the total number of nurses was 5,742, the sample sizes ranging from 46 to 3,409. In qualitative studies, the total number of nurses was 73 and the sample sizes ranged from 6 to 52. Other participant groups included nurse leaders (*N* = 405), strategic healthcare managers (*N* = 10), dental students (*N* = 196), clinical faculty members (*N* = 41), regents and officers (*N* = 19), and subscribers to the digital newsletter of three widely read nursing journals in the Netherlands (*N* = 49). One study used a combination of healthcare professionals (*N* = 118) without specifying the participant groups. Response rates were indicated in 5 (63%) studies using quantitative data, and they ranged from 16% to 73% (Table 1).

#### 4.4.3 Sampling

The authors used both nonprobability sampling methods, including purposive sampling (*N* = 5) and convenience sampling (*N* = 1) and probability sampling methods, including stratified sampling (*N* = 1), strategic sampling (*N* = 1), and systematic sampling (*N* = 1). The sampling method was not indicated in five studies (Table 1).

#### 4.4.4 Data collection

The authors used instruments that they had developed themselves to measure ethical competence and to collect the data in all quantitative studies. In three studies (Asahara et al., 2015, 2013 ; Chambers, 2011), the structure of the instrument followed the Four‐component model for determining moral behaviour described by Rest (1994). Other instruments were developed based on interviewing (Cusveller & Schep‐Akkerman, 2016; Jormsri et al., 2004), literature reviewing (Jormsri et al., 2004; Poikkeus, Numminen, et al., 2014; Poikkeus et al., 2016) and deductive reasoning based on the literature (Poikkeus et al., 2016).

Semistructured interviews (*N* = 5), in‐depth interviews (*N* = 2), and ethnographic participant observation (*N* = 1) were used for data collection in qualitative studies. All literature reviews and the concept analysis study retrieved the data from relevant databases while the number of papers reviewed/analysed ranged from 18‐89.

#### 4.4.5 Data analysis

The data analysis methods varied based on the designs applied in different studies. The majority of the studies used statistical analysis methods, followed by content analysis. Description of the data analysis process was not provided or the description was vague in three studies (Cusveller & Schep‐Akkerman, 2016; Lechasseur et al., 2016; Schaefer & Vieira, 2015).

### 4.5 Reliability and validity assessments

The reliability and validity assessments of the studies were addressed at different levels of sophistication. In quantitative studies, internal consistency using Cronbach's alpha was the most commonly stated (*N* = 6) reliability assessment. Other commonly described assessments referring to reliability and validity were face validity (*N* = 5), content validity (*N* = 5), and piloting (*N* = 5). Two studies established the construct validity of the instrument used and stability of the results. None of the studies used power analysis to determine sophisticated sample size.

In qualitative studies as well as in literature reviews and concept analysis studies, researcher validation was the most commonly addressed validity assessment (*N* = 4), followed by saturation of the data (*N* = 3). One study used a specific criterion (credibility, confirmability, dependability, transferability) to address the validity of the study. General discussion about study validity without any specific criterion was provided in six studies. Six studies provided no reliability or validity assessments, or the discussion on these matters was vague (Cusveller & Schep‐Akkerman, 2016; Höglund et al., 2010; Lechasseur et al., 2016; Peter et al., 2015; Schaefer & Vieira, 2015).

Most commonly, limitations of the studies dealt with low response rates, sampling/participant biases, and limitations to the instruments. Attention was also paid to self‐report bias, lack of generalization, and social desirable bias. Six studies provided no discussion about the study limitations (Table 2).

**Table 2:** Validity and reliability assessments and limitations of the studies

Author	Quantitative							Qualitative							Limitations											
	Face validity	Content validity	Construct validity	Internal consistency	Stability	Piloting	Power analysis	Credibility	Confirmability	Dependability	Transferability	Saturation	Researcher validation	Discussion about validity/trustworthiness without any specific criteria	Small sample size	Low response rate	Sampling bias/Participant bias	Social desirable bias	Self‐report bias	Limitations to the instrument	Limited scope	Lack of generalization	Language bias	Publication bias	Data analysis process	No limitation report
Quantitative studies																										
Jormsri et al. (2004)	+	+/E/CVI	‐	+	‐	+	‐	‐	‐	‐	‐	‐	‐	‐	‐	‐	‐	‐	‐	‐	‐	‐	‐	‐	‐	+
Chambers (2011)	+	+/E	‐	+	+	+	‐	‐	‐	‐	‐	‐	‐	‐	‐	‐	‐	‐	‐	‐	‐	‐	‐	‐	‐	+
Asahara et al. (2013)	+	+/E	+/CFA/EFA	+	+	‐	‐	‐	‐	‐	‐	‐	‐	‐	‐	+	‐	‐	‐	+	+	‐	‐	‐	‐	‐
Poikkeus, Numminen, et al., 2014	‐	‐	‐	+	‐	+	‐	‐	‐	‐	‐	‐	‐	‐	‐	+	‐	+	‐	+	‐	‐	‐	‐	‐	‐
Asahara et al. (2015)	+	+/E	+/CFA/EFA	+	‐	‐	‐	‐	‐	‐	‐	‐	‐	‐	‐	+	‐	‐	‐	+	‐	‐	‐	‐	‐	‐
Cusveller and Schep‐Akkerman (2016)	‐	‐	‐	‐	‐	‐	‐	‐	‐	‐	‐	‐	‐	‐	‐	+	+	‐	+	‐	‐	‐	‐	‐	‐	‐
Poikkeus et al. (2016)	+	+/E/CVI	‐	+	‐	+	‐	‐	‐	‐	‐	‐	‐	‐	‐	+	‐	‐	+	+	‐	‐	‐	‐	‐	‐
Qualitative studies																										
Höglund et al. (2010)	‐	‐	‐	‐	‐	‐	‐	‐	‐	‐	‐	+	‐	‐	+	‐	+	‐	‐	‐	‐	‐	‐	‐	‐	‐
Cusveller (2012)	‐	‐	‐	‐	‐	‐	‐	‐	‐	‐	‐	+	+	+	‐	‐	‐	‐	‐	‐	‐	‐	‐	‐	‐	+
Peter et al. (2015)	‐	‐	‐	‐	‐	‐	‐	‐	‐	‐	‐	‐	+	‐	‐	‐	+	+	‐	‐	‐	‐	‐	‐	‐	‐
Barkhordari‐Sharifabad et al. (2016)	‐	‐	‐	‐	‐	‐	‐	+	+	+	+	+	‐	+	‐	‐	‐	‐	‐	‐	‐	‐	‐	‐	‐	+
Falkenström et al. (2016)	‐	‐	‐	‐	‐	‐	‐	‐	‐	‐	‐	‐	+	+	‐	‐	‐	‐	‐	‐	‐	+	‐	‐	‐	‐
Mixed‐methods studies																										
Molewijk, Verkerk, et al. (2008)	‐	‐	‐	‐	‐	+	‐	‐	‐	‐	‐	‐	+	+	‐	‐	‐	‐	‐	‐	‐	‐	‐	‐	‐	+
Literature reviews and Concept analysis																										
Poikkeus, Leino‐Kilpi, et al., 2014	‐	‐	‐	‐	‐	‐	‐	‐	‐	‐	‐	‐	+	+	‐	‐	+	‐	‐	‐	‐	‐	+	+	‐	‐
Schaefer and Vieira (2015)	‐	‐	‐	‐	‐	‐	‐	‐	‐	‐	‐	‐	+	‐	‐	‐	‐	‐	‐	‐	‐	‐	‐	‐	‐	+
Kulju et al. (2016)	‐	‐	‐	‐	‐	‐	‐	‐	‐	‐	‐	‐	+	+	‐	‐	‐	‐	‐	‐	‐	‐	‐	‐	+	‐
Lechasseur et al. (2016)	‐	‐	‐	‐	‐	‐	‐	‐	‐	‐	‐	‐	‐	‐	‐	‐	‐	‐	‐	‐	‐	+	‐	‐	‐	‐

Note

+: reported; ‐: not reported or unclear; E: expert panel; CVI: content validity index; EFA: exploratory factor analysis; CFA: confirmatory factor analysis.

## 5 DISCUSSION

Ethical competence is a precondition for quality health care. Healthcare reforms, development of new technology, and allocation of resources pose several challenges to healthcare professionals’ ethical competence. To maintain and promote high‐quality ethical care, several international and national guidelines have been published (Deshpande, Joseph, & Prasad, 2006; ICHRN, 2010). All these guidelines emphasize the need for research in the field of ethical competence.

This review identified a limited amount of research in the field of ethical competence. However, the interest toward ethical competence seems to be increasing as the majority of the studies were published recently. This increasing interest might be explained by the emphasis on ethical environment (Lin et al., 2013) and ethical integrity (Eby, Hartley, Hodges, & Hoffpauir, 2017). Furthermore, current ethically charged issues, such as priority setting (Norheim, 2016) and care rationing (Rooddehghan, Yekta, & Nasrabad, 2016), are evoking ethical concerns. Recognition of the role of ethical competence in managing these might have contributed to the increase in interest in this research area.

Ethical competence can be approached from three focus areas: conceptualization, measuring, and realization of ethical competence. The emphasis of the research seems to be on the first and the second. This is natural, as the conceptualization of ethical competence is a rather recent phenomenon (Jormsri et al., 2004), there are not many research groups interested in the topic and research base and theoretical understanding develops slowly. Furthermore, the three focus areas still fail to form a homogenous picture of the research area, leaving it scattered.

Theoretically and empirically, ethical competence seems to be a multidefinitional concept lacking a convergent understanding of its definition and construction. This is understandable as the whole research area is limited and at its early stages. Furthermore, competence in itself is often considered a difficult combination of knowledge, skills, and attitudes (Stoof, Martens, Merriënboer, & Basties, 2002), not to mention ethical competence, where the term “ethical” also entails a complicated mix of content areas. This makes the conceptualization even more complicated. However, the theoretical base of the concept has been seen as sufficient as a transition phase from conceptualization to measuring ethical competence is clearly ongoing.

Some instruments measuring ethical competence were identified. All instruments were national ones and in the early stages of their development process, requiring further research to confirm their psychometric properties and validation. So far, the development processes have been rather slow, albeit systematic, using different statistical approaches. However, these different statistical approaches challenge the unambiguous methodological quality comparisons of the instruments. Measuring ethical concepts, which are often abstract, is complex, making the slow progress understandable. This review revealed the fact that ethical competence can be measured subjectively, and the instruments found in this review can serve as a starting point for further research. Furthermore, juxtaposing two or more instruments could be considered.

Methodologically, the research was rather heterogeneous. However, descriptive design predominated both quantitative and qualitative research approaches. To gain a deeper understanding of ethical competence, different multidimensional designs are needed. Intervention studies using educational interventions may offer a possibility to have an impact on the ethical competence of healthcare professionals (Stolt, Leino‐Kilpi, Ruokonen, Repo, & Suhonen, 2018). Most commonly, studies reported low‐response rates and sampling/participant bias as their limitations. More attention should be paid on sampling procedures to tackle these issues and to recruit samples with generalizable results (Suhonen, Stolt, Katajisto, & Leino‐Kilpi, 2015). However, these limitations do not concern only research on ethical competence; they seem to be very common in many areas of healthcare research, especially in research on empirical ethics (Koskenvuori, Numminen, & Suhonen, 2017; Suhonen, Stolt, Virtanen, & Leino‐Kilpi, 2011).

Research on ethical competence seems to be focusing on nurses. This is interesting, as the codes of ethics have been used in all professional groups. Although nurses form the largest professional group in clinical practice (WHO., 2006), it would be beneficial to study ethical competence of other healthcare professionals as care is predominantly multiprofessional. Furthermore, it is important to continue the research among healthcare managers. Ethical competence of managers relates to ethical sensitivity and the ability to identify and solve ethical problems among employees (Poikkeus et al., 2016).

Colleagues and leaders were determined to have a key role in enhancing ethical competence (Poikkeus, Leino‐Kilpi, et al., 2014). Furthermore, moral competencies could be improved through moral case deliberations (Molewijk, Verkerk, et al., 2008). This knowledge provides a good start, but more research on these issues is needed. In particular, what are the strategies and interventions that healthcare organizations could use to strengthen ethical competencies of their employees? Furthermore, research on the growth of healthcare professionals’ ethical competence could provide new insights and understanding of this multidimensional phenomenon.

### 5.1 Strengths and limitations

This scoping review followed a predetermined systematic protocol (Arksey & O'Malley, 2005). The data were retrieved from six international scientific databases in the field of health sciences and philosophy. The literature search produced a large number of hits and overlap between the databases was evident as indicated by the high number of duplicates (*N* = 539), which were removed in the first phase. Although Medline covers a wide range of research in health sciences (Bahaadinbeigy, Yogesan, & Wootton, 2006), it is recommended to use other databases as well to ensure comprehensiveness of the search (Seaton, 2006). The search terms used in this review were on general level, such as ethical/moral competence and ethical/moral skills. This wide approach led to a multitude of information, which is desirable in scoping reviews (Davis, Drey, & Gould, 2009).

Although research quality evaluation is not an initial part in scoping reviews, we performed critical appraisal of the studies using international evaluation criteria (Joanna Briggs Institute, 2017). A suitable evaluation tool was selected according to each study design. The use of these tools was not without problems. The methodological quality of the studies varied, leading to uncertainty as to which methods were used in these particular studies. To overcome this uncertainty, quality evaluations were conducted within the research group where each researcher evaluated six studies. These evaluations were cross checked within the group and consensus was achieved. Based on quality evaluations, no studies were excluded which might have led to incomplete data synthesis and findings. However, we aimed to gather a comprehensive perspective to ethical competence and therefore including all studies is reasonable.

Analysis of the studies was started by tabularization of the descriptive information and main findings. This work sheet was designed for the purposes of this review to ensure the focus on key issues. The terms and sentences used by the original authors were used as they appeared in the text, and no interpretations were made.

## 6 CONCLUSIONS

Ethical competence is an ultimate necessity to guarantee high‐quality health care in the future. Research in this field is limited but seems to be increasing. The focus areas of the research can be classified into the following three: conceptualization, measuring, and realization of the ethical competence. The focus areas still do not seem to form a homogeneous picture of the research area, leaving it scattered. Conceptualization of ethical competence is rather new and the definitions and constructions provided vary. However, the theoretical base of the concept has been seen as sufficient as the research seems to be in a transition phase from theorization to empirical measurement. Thus, the measurements identified need more validations. Methodologically, the research is rather heterogeneous. To gain a deeper understanding, multidimensional research designs are needed. Furthermore, issues affecting generalizability of the research results need more attention. Research in this area is nurse oriented, but it would be beneficial to expand the research to other healthcare professionals as well. In addition, research on the improvement, enhancement, and growth of ethical competence is needed. These can be used to support healthcare reforms and to promote quality in health care.

## CONFLICT OF INTEREST

Authors declare no conflicts of interest.

## AUTHOR CONTRIBUTION

All authors have agreed on the final version and meet at least one of the following criteria [recommended by the ICMJE (https://www.icmje.org/recommendations/ )]:

- substantial contributions to conception and design, acquisition of data or analysis and interpretation of data;
- drafting the article or revising it critically for important intellectual content.
